# Relationship Between Disease Severity and Resting Electrocardiograms of Adults With Sickle Cell Anemia in a Tertiary Institution in Southern Nigeria

**DOI:** 10.7759/cureus.15296

**Published:** 2021-05-28

**Authors:** Mercy O Dic-Ijiewere, Airenakho Emorinken, Austine O Obasohan, Peter O Okokhere, Ebenezer O Dic-Ijiewere, Odianosen S Otumu

**Affiliations:** 1 Department of Medicine, Irrua Specialist Teaching Hospital, Irrua, NGA; 2 Department of Medicine, College of Medical Sciences, University of Benin, Benin City, NGA; 3 Department of Chemical Pathology, Ambrose Alli University, Ekpoma, NGA; 4 Department of Hematology, Irrua Specialist Teaching Hospital, Irrua, NGA

**Keywords:** adult sickle cell anemia, disease severity, electrocardiography, cardiac morbidities

## Abstract

Introduction

Sickle cell anemia (SCA) in adults has many clinical manifestations. These manifestations are due to effects of recurrent hemolysis, anemia, and ischemia-reperfusion injury on various organs, including the heart. These factors determine the severity of the disease.

Objectives

The aim of the study was to assess the severity of SCA using a scoring system consisting of clinical and laboratory parameters. In addition, the study aimed to determine the electrocardiographic abnormalities in the adult SCA population.

Study design

This was a cross-sectional, observational study conducted in the medical outpatient clinic of Irrua Specialist Teaching Hospital, Irrua, Nigeria.

Methodology

Sixty SCA patients who were older than 18 years old were recruited for this study between February 2017 and January 2018. Sixty healthy individuals matched for age and sex were recruited to serve as controls. Patients who were pregnant or having an acute crises were excluded from the study. Each participant had an electrocardiogram and a SCA severity score was calculated using their clinical history and complete blood count. Data analysis was carried out using the IBM Statistical Package for Social Sciences Statistics® software, version 21 (IBM SPSS Statistics for Windows, Armonk, NY) and statistical significance assigned to p-values less than 0.05.

Results

Severity scores for SCA ranged between 7 and 24, with a mean score of 14.5 ± 4.04. Out of the 60 patients, 14 (23.3%), 39 (65%), and seven (11.7%) participants met criteria for mild, moderate, and severe disease, respectively. Tachycardia, prolonged QTc, and the presence of ST-segment and T-wave abnormalities were significantly associated with severe SCA (p = 0.024, p = 0.027, and p = 0.018, respectively). There was positive correlation between SCA severity scores and P-wave duration (r = 0.327, p = 0.011), QRS dispersion (r = 0.298, p = 0.021), QTc interval (r = 0.332, p = 0.010), and QTc dispersion (r = 0.320, p = 0.013).

Conclusion

This study demonstrated that moderate and severe forms of SCA are common in our region. Tachycardia, left atrial abnormality, prolonged corrected QT interval, and the presence of ST-segment and T-wave changes are electrocardiographic findings associated with more severe forms of the disease. These abnormalities are significant etiologies of cardiac morbidity and mortality in SCA.

## Introduction

Sickle cell anemia (SCA) is the most common form of sickle cell disease (SCD) worldwide [1]. It occurs as a result of a homozygous inheritance of abnormal hemoglobin known as sickle hemoglobin (HbS). The disease is characterized by acute periods of ill-health (called crises) and periods of relative well-being [2]. Recurrent hemolysis, leading to chronic anemia and recurrent vaso-occlusion with ischemia-reperfusion injury to multiple organs, are the hallmark of the disease [2-3].

SCA causes abnormalities in the function of the heart and other organs. Cardiac features, such as fatigue, dyspnea on exertion, palpitations, mild lower extremity swelling, chest pain, tachycardia, wide pulse pressure, presence of distended neck veins, displaced apex beat, and presence of murmurs, initially occur from anemia [4-5]. Dyspnea at rest, orthopnea, and extensive lower limb swelling may be associated with severe anemia [6]. Cardiovascular complications, such as reduced exercise capacity, cardiac arrhythmias, pulmonary hypertension, diastolic dysfunction, and heart failure, may also manifest with the above symptoms.

Apart from anemia, recurrent hemolysis, recurrent vaso-occlusion, and iron overload can adversely affect cardiac function. These effects are mediated by the depletion of nitric oxide levels in pulmonary blood vessels due to high free hemoglobin levels, deposition of excess unbound iron within myocytes, vaso-occlusion within myocardial blood vessels, and coronary vasospasm from the release of inflammatory mediators. Coronary vasospasm and myocardial vaso-occlusion can cause myocardial ischemia [7-9].

Electrocardiography (ECG) is an affordable and relatively accessible non-invasive procedure used to assess cardiac structure and function. Several studies have observed abnormal electrocardiograms in SCA patients [10-14]. Abnormalities reported in these studies include tachycardia, left ventricular hypertrophy, left atrial enlargement, right ventricular hypertrophy, ST-segment and T- wave abnormalities, arrhythmias (like atrial and ventricular premature complexes), prolonged corrected QT interval, and first-degree atrioventricular block [11-14]. Significantly higher mean heart rate, P-wave duration, P-wave dispersion, PR interval, QRS duration, QRS dispersion, QTc interval, and QTc dispersion in SCA subjects, when compared to normal controls, have also been observed [12].

However, studies have not been carried to establish a relationship between the presence of these abnormalities and the severity of SCA. This may be because there is no universal system used to determine the severity of SCA. Several studies have adopted different models based on genetic factors and the phenotypic expression of the disease, such as the frequency of crises and hospital admissions, degree of anemia and frequency of blood transfusions, and the presence of complications, to determine the severity of the disease [15-20]. None of these studies have matched the severity profiles of patients with cardiac manifestations of SCA.

As significant proportions of SCA patients survive into adulthood due to improved health care services, it is known that cardiovascular comorbidities contribute significantly to the complications in SCA and contribute to mortality [21]. Hence, the objective of this study was to assess the cardiac function of SCA subjects using an easily assessable tool (ECG) and to determine if there is any relationship between the findings and the severity of SCA assessed by clinical and laboratory parameters.

## Materials and methods

This was an observational cross-sectional study conducted between February 2017 and January 2018 in the medical outpatient clinic of Irrua Specialist Teaching Hospital (ISTH) Irrua, a federal government-owned tertiary institution located in Edo State, South South Nigeria. The study was commenced after approval of the Health Research Ethics Committee (HREC) of the hospital (ISTH/HREC/2016/June/035).

The study population consisted of 120 adults (50 males and 70 males). Sixty of them were SCA patients, while 60 were healthy age and sex-matched controls.

The selected SCA patients had been diagnosed by electrophoresis and were on follow-up at the facility. The controls were apparently healthy individuals who were hemoglobin AA diagnosed by electrophoresis. Pregnant women and SCA patients in acute crises were excluded from the study. 

Demographic data including age, gender, ethnicity, religion, level of education, and occupation were obtained. Medical information was obtained from each patient (number of admissions for sickle cell crises in the past year, number of blood transfusions in the last year, and presence of symptoms and complications, as described by Ballas et al. in the Sickle Cell Disease Cooperative Study [15]).

Hemoglobin concentration, white cell count, reticulocyte count, and unconjugated bilirubin concentration were assessed. Hemoglobin concentration and white cell count were measured using the Sysmex XP-300™ Automated Hematology Analyzer (Sysmex Corp., Kobe, Japan). Reticulocyte count was assessed on a microscope by counting the number of reticulocytes seen in a thin film of blood mixed with new methylene blue. It was expressed as a percentage of the total red blood cells seen on the blood film. Unconjugated bilirubin was assessed using the SP-113 Visible Spectrophotometer (Axiom Solutions, Bürstadt, Germany).

The severity of SCA was assessed using the scoring system of Adegoke and Kuti [18] as shown in Table 1. SCA was graded as a mild disease if the severity score was less than 8, moderate if the score was between 8 and 17, and severe if the score was greater than 17.

**Table 1:** Sickle Cell Anemia Severity Scoring System ACS: acute chest syndrome; AVN: avascular necrosis; CVD: cerebrovascular disease

Parameter and score
1. For number of painful episodes in the previous 12 months, score:
a. 0 when the number is 0
b. 1 when the number is 1
c. 2 when the number is 2 or 3
d. 3 when the number is > 3
2. For number of transfusions in the previous 12 months, score:
a. 0 when the number is 0
b. 1 when the number is 1
c. 2 when the number is 2 or 3
d. 3 when the number is > 3
3. For number of hospitalizations in the previous 12 months, score:
a. 0 when the number is 0
b. 1 when the number is 1
c. 2 when the number is 2 or 3
d. 3 when the number is > 3
4. For liver enlargement, score:
a. 0 when < 2 cm
b. 1 when 2 to 5 cm
c. 2 when > 5 cm
5. For splenic enlargement, score:
a. 0 when < 5 cm
b. 1 when 5 to 10 cm
c. 2 when > 10 cm
6. For packed cell volume, score:
a. 0 when ≥ 24%
b. 1 when 18% – 23%
c. 2 when < 18%
7. For white blood cell count, score:
0 when < 11,000/mm^3^
b. 1 when between 11,000 and 15,000/mm^3^
c. 2 when > 15,000/mm^3^
8. For lifetime cumulative incidence of specific complications, score:
a. 5 when CVD is/was present, 0 when absent
b. 3 when ACS is/was present, 0 when absent
c. 3 when pneumococcal meningitis is/was present, 0 when absent
d. 2 when AVN is present, 0 when absent
e. 1 each when gall stone, chronic leg ulcer, osteomyelitis, or priapism is/was present, 0 when absent.

Resting 12-lead ECG was performed on all subjects using the Edan electrocardiograph machine model #ECG-1350K Cardiofax, (Nihon Kohden Corp., Tokyo, Japan) at a paper speed of 25 mm/s and standardized at 0.1 mV/mm. The heart rate, cardiac axis, P-R interval, QRS duration, and QT interval were measured. Heart rate correction for the QT interval was performed using Bazett’s formula (QTc = QT/√RR) [22]. The dispersion of P-wave, QRS, and QTc intervals were measured as the differences between the maximum and minimum values of each of these parameters on the 12-lead ECG.

The data obtained were entered and analyzed using the commercially available IBM Statistical Package for Social Sciences (SPSS) Statistics® 2012, version 21.0 for Windows (IBM SPSS Statistics, Armonk, NY). Normally distributed continuous data were described using mean and standard deviation. Categorical data were described using frequencies, percentages, and proportions. Differences in means across the three levels of severity were analyzed using ANOVA (analysis of variance). Pearson’s correlation test was used to assess the relationships between SCA severity scores and electrocardiographic parameters. P-value < 0.05 was considered as statistically significant.

## Results

The age range of study participants was 18 to 35 years. The gender distribution and overall mean age of the SCA patients and controls were comparable. There were significant differences in the hematological and electrocardiographic parameters of both groups. This is shown in Table 2.

**Table 2:** Comparison of Epidemiological, Hematological, and Electrocardiographic Parameters of Sickle Cell Anemia Subjects and Controls * Statistically significant at p < 0.05 # Analyzed by Chi-square statistic bpm: beats per minute; disp: dispersion; dur: duration; Hb conc: hemoglobin concentration; HR: heart rate; int: interval; msec: milliseconds; QTc: corrected QT interval; retic: reticulocyte; SD: standard deviation; unconj bil: unconjugated bilirubin; WBC: white blood cell

Variable	Case - Mean ± SD/Frequency (%)	Control - Mean ± SD/Frequency (%)	P-value
Mean age	24.40 ± 4.43	24.38 ± 3.60	0.953
Gender^#^			
Male	25 (41.7)	25 (41.7)	1.000
Female	35 (58.3)	35 (58.3)	
Hb conc	7.58 ± 0.43	13.01 ± 1.77	< 0.001*
WBC count	14660 ± 2005.18	4685 ± 1357.57	< 0.001*
Retic count	2.30 ± 0.36	0.52 ± 0.16	< 0.001*
Unconj bil	1.53 ± 0.33	0.47 ± 0.08	< 0.001*
P-wave dur (msec)	123.00 ± 116.59	101.35 ± 5.28	0.153
P-wave disp (msec)	30.83 ± 24.24	9.58 ± 4.72	< 0.001*
PR int (msec)	166.82 ± 20.72	157.42 ± 14.43	0.005*
QRS dur (msec)	90.87 ± 10.16	89.70 ± 5.93	0.444
QRS disp (msec)	23.08 ± 8.74	7.08 ± 3.71	< 0.001*
QT dur (msec)	363.57 ± 32.81	380.37 ± 20.36	< 0.001*
QTc dur (msec)	434.47 ± 24.04	414.85 ± 12.56	< 0.001*
QTc disp (msec)	40.20 ± 18.33	21.34 ± 3.95	< 0.001*

SCA severity scores in this study ranged between 7 and 24, with a mean of 14.5 ± 4.04. Table 3 shows the differences in the pattern of the severity of SCA between the sexes and across the age groups. The majority of the study subjects (65%) had severity scores for moderate SCA. There was no significant difference in SCA severity between gender and across the age categories.

**Table 3:** Severity of Sickle Cell Anemia Between Gender and Across Age Groups ^0^ Expressed as frequency (%) and analyzed with Fischer's test ^+^ Expressed as mean ± SD and analyzed using independent samples t-test SD: standard deviation

Parameter	Mild – Mean ± SD/ Frequency (%)	Moderate – Mean ± SD/ Frequency (%)	Severe – Mean ± SD/ Frequency (%)	P-value
Age (years)^0^				
18 - 22	7 (11.6)	13 (21.7)	2 (3.3)	0.70
23 - 27	6 (10.0)	14(23.3)	3 (5.0)	
28 - 32	1 (1.7)	8 (13.3)	1 (1.7)	
33 - 37	0 (0.0)	4 (6.7)	1 (1.7)	
Mean age (years)^+^	(22.14 ± 2.98)	(23.83 ± 3.06)	(25.28 ± 4.77)	0.068
Gender^0^				
Male	4 (6.6)	18 (30.0)	3 (5.0)	0.423
Female	10 (16.7)	21 (35.0)	4 (6.7)	

Electrocardiographic parameters at each level of severity are also shown in Table 4. Their mean values were observed to increase with each severity group, apart from the PR interval. These findings were statistically significant for only the QTc interval and QTc dispersion (p = 0.003 and p = 0.002, respectively). The mean reticulocyte count was significantly lower in the group with mild severity scores and highest in the group with severe SCA severity scores. The mean unconjugated bilirubin level was not observed to change across the severity groups.

**Table 4:** Electrocardiographic Parameters in Sickle Cell Anemia Severity Groups * statistically significant at p < 0.05 bpm: beats per minute; disp: dispersion; dur: duration; HR: heart rate; int: interval; msec: milliseconds; QTc: corrected QT interval; retic: reticulocyte; SD: standard deviation

Parameter	Severity of Sickle Cell Anemia			
	Mild (Mean ± SD)	Moderate (Mean ± SD)	Severe (Mean ± SD)	P-value
HR (bpm)	88.18 ± 11.62	89.17 ± 16.62	89.50 ± 10.07	0.745
P-wave dur (msec)	104.00 ± 9.21	117.33 ± 14.77	126.08 ± 130.28	0.905
P-wave disp (msec)	21.67 ± 5.16	28.33 ± 9.31	32.39 ± 26.68	0.586
PR int (msec)	171.83 ± 9.47	165.08 ± 22.19	175.67 ± 13.98	0.417
QRS dur (msec)	90.29 ± 7.33	91.23 ± 11.37	89.83 ± 8.23	0.927
QRS disp (msec)	22.50 ± 8.09	23.93 ± 10.41	25.00 ± 10.00	0.748
QTc interval (msec)	422.17 ± 30.81	434.50 ± 23.24	446.50 ± 20.85	0.003*
QTc disp (msec)	28.00 ± 10.06	42.54 ± 19.95	50.33 ± 18.57	0.002*
Retic count (%)	1.95 ± 0.11	1.98 ± 0.36	2.23 ± 0.49	0.043*
Unconjugated bilirubin (mg/dL)	1.65 ± 0.16	1.5 ± 0.35	1.72 ± 0.21	0.210

Table 5 shows that electrocardiographic abnormalities were present in all severity groups. Sinus arrhythmias, left atrial abnormality, left anterior fascicular block, prolonged QTc interval, and ST-segment and T-wave abnormalities occurred in higher proportions of those who had moderate and severe SCA severity scores. As the severity of SCA increased, the proportion of SCA patients with abnormal ECG tracings also increased. All patients with severe disease had abnormal ECG tracings. Sinus tachycardia, prolonged QTc, and presence of ST-segment and T-wave abnormalities occurred in significant proportions of the study subjects with moderate and severe SCA severity scores.

**Table 5:** Electrocardiography Abnormalities Observed at Different Levels of Sickle Cell Anemia Severity * statistically significant at p < 0.05 AV: atrioventricular; ECG: electrocardiography; QTc: corrected QT interval

ECG Abnormality	Severity of Sickle Cell Anemia			
	Mild (n = 14) Frequency (%)	Moderate (n = 39) Frequency (%)	Severe (n = 7) Frequency (%)	P-value
Sinus tachycardia	6 (42.9)	4 (10.2)	2 (28.6)	0.024*
Sinus arrhythmia	6 (42.9)	11 (27.5)	0 (0.0)	0.147
Abnormal axes	2 (14.3)	6 (15.3)	3 (42.9)	0.411
Left atrial abnormality	3 (21.4)	9 (23.1)	3 (42.9)	0.328
Right atrial abnormality	1 (7.1)	1 (2.6)	1 (14.3)	0.630
Left ventricular hypertrophy	3 (21.4)	12 (30.8)	2 (28.6)	0.796
Right ventricular hypertrophy	2 (14.3)	5 (12.8)	1 (14.3)	0.597
First-degree AV block	0 (0.0)	2 (5.1)	1 (14.3)	0.596
Right bundle branch block	2 (14.3)	3 (7.7)	1 (14.3)	0.597
Left anterior fascicular block	0 (0.0)	1 (2.6)	1 (14.3)	0.144
Non-specific intraventricular conduction abnormality	0 (0.0)	3 (7.7)	1 (14.3)	0.343
ST-segment & T-abnormality	2 (14.3)	7 (17.9)	4 (57.1)	0.018*
Prolonged QTc	2 (14.3)	8 (20.5)	4 (57.1)	0.027*
Frequency of abnormal ECGs	8 (57.1)	27 (69.2)	7 (100.0)	0.165

This study observed a weak, but positive, correlation between the severity scores of SCA and age (in years), P-wave duration, P-wave dispersion, QTc interval, and QTc dispersion (Table 6). Reticulocyte count had a moderate positive correlation with severity scores for SCA. Male gender and unconjugated bilirubin concentration had no correlation with the severity of SCA. 

**Table 6:** Correlates of Sickle Cell Anemia Severity * statistically significant msec: milliseconds; QTc: corrected QT interval

Variable	Correlation coefficient	P-value
Age (in years)	0.291	0.020*
Male gender	-0.042	0.748
P wave duration (msec)	0.327	0.011*
P wave dispersion (msec)	0.272	0.036*
QRS duration (msec)	-0.250	0.852
PR interval (msec)	0.048	0.715
QRS dispersion	0.298	0.021*
QTc duration (msec)	0.332	0.010*
QTc dispersion (msec)	0.347	0.007*
Reticulocyte count (%)	0.442	0.000*
Unconjugated bilirubin (mg/dl)	0.057	0.664

## Discussion

This study has stratified adult SCA subjects into severity groups using a scoring system made up of clinical and laboratory parameters. This study also showed that abnormal electrocardiograms were seen in almost two-thirds (39 out of 60) of SCA subjects. The proportion of SCA subjects with abnormal electrocardiograms increased as the severity of the disease increased.

Using the scoring system adapted for the study, the majority (65%) of adult SCA subjects had severity scores for moderate disease. This is similar to studies carried out in Ibadan and Lagos [16, 20]. The proportions observed in those studies may not be comparable to those observed in this study due to the differences in parameters used for assessing disease severity.

However, in comparison to the study of Adegoke and Kuti, the proportions are similar as both studies used the same method to assess the severity of SCA (23.3%, 65%, and 11.7% for this study and 23.9%, 55.7%, and 10.4% in the Adegoke and Kuti study) [18].

This study observed higher mean P-wave duration, P-wave dispersion, QRS dispersion, QTc interval, and QTc dispersion as the SCA severity increased. These changes were statistically significant for QTc interval and QTc dispersion (p = 0.003 and p = 0.002, respectively). Previous studies have established that these findings occur in higher proportions of SCA subjects when compared to normal controls [11-13, 23].

Corrected QT (QTc) interval is a measure of the time taken for ventricular activation and recovery. Although the QTc interval can be affected by various factors, like changes in autonomic tone, hypocalcemia, hypokalemia, drugs, and the presence of myocardial ischemia among others, it (alongside prolonged QTc dispersion) is implicated in ventricular arrhythmias as predictors of sudden death and mortality in individuals with ischemic heart disease, heart failure, and in apparently healthy individuals [24-25].

This study observed that tachycardia, prolonged QTc, and ST-segment and T-wave abnormalities (an indicator of myocardial ischemia) occurred mainly in SCA subjects with moderate and severe disease. These findings indicate that moderate to severe disease may be associated with a higher risk of cardiac arrhythmias and sudden death.

Prolonged QTc interval has also been identified as a risk factor for sudden death in SCA patients and is strongly associated with complications of SCA, such as severe anemia requiring multiple blood transfusions, heart failure, pulmonary hypertension, and sudden death [21, 26-28].

There was a significant positive correlation between the SCA severity scores and P-wave duration, P-wave dispersion, QRS dispersion, QTc interval, and QTc dispersion. These findings may suggest that severe SCA is associated with cardiovascular abnormalities like left atrial abnormality, intraventricular conduction abnormalities, increased risk of arrhythmias, and sudden death.

## Conclusions

This study demonstrated that moderate and severe forms of SCA are common in Southern Nigeria. Our study shows that electrocardiographic abnormalities, such as tachycardia, left atrial abnormality, prolonged corrected QT interval, ST-segment abnormalities, and T-wave changes (known to be strongly associated with myocardial ischemia, risk of cardiac arrhythmias and sudden death) occur in significant proportions of adults with moderate to severe forms of SCA.
